# Carbolic Acid

**Published:** 1870-01

**Authors:** 


### Selections.

Carbolic Acid.—A short article on the employment of carbolic acid in surgery, published in the *Bulletin General de Therapeutique Medicale et Chirurgicale*, shows that authorities on the continent do not regard it with quite as much favor as some British and American surgeons do. Since, says our French cotemporary with much justice, carbolic acid has become the fashion, it has been used, or rather abused, by attributing to it impossible properties; it obtains these marvellous properties, above all, in the hands of English surgeons, who are infatuated with it. Our French authority holds that in spite of the great powers attributed to it by Mr. Lister, the death-rate has been greater in Mr. Lister's hospital since its employment than it was some years ago before it was known; he maintains that the cases recorded of excision of the wrist, of fracture opening the ankle-joint, &c., cured without suppuration, are but exceptions such as every surgical method can claim; that such is the case to some extent seems clear from the following opinion of the merits of carbolic acid published by Dr. J. Bell:

"The cases I have reported are merely examples out of the many we have had, illustrating the same great practical fact, that the use of carbolic acid dressing as an antiseptic method, followed out with due care, and with the great precautions which are essential to success, is able in some cases practically to get rid of suppuration; in nearly every case greatly to diminish its quantity, and entirely to destroy its fetor."

